# Bayesian networks for network inference in biology

**DOI:** 10.1098/rsif.2024.0893

**Published:** 2025-05-07

**Authors:** James Hammond, V. Anne Smith

**Affiliations:** ^1^ Department of Biology, University of Oxford, Oxford, UK; ^2^ School of Biology, University of St Andrews, St Andrews, UK

**Keywords:** Bayesian networks, network inference, computational neuroscience, machine learning, biological networks, biological data

## Abstract

Bayesian networks (BNs) have been used for reconstructing interactions from biological data, in disciplines ranging from molecular biology to ecology and neuroscience. BNs learn conditional dependencies between variables, which best ‘explain’ the data, represented as a directed graph which approximates the relationships between variables. In the 2000s, BNs were a popular method that promised an approach capable of inferring biological networks from data. Here, we review the use of BNs applied to biological data over the past two decades and evaluate their efficacy. We find that BNs are successful in inferring biological networks, frequently identifying novel interactions or network components missed by previous analyses. We suggest that as false positive results are underreported, it is difficult to assess the accuracy of BNs in inferring biological networks. BN learning appears most successful for small numbers of variables with high-quality datasets that either discretize the data into few states or include perturbative data. We suggest that BNs have failed to live up to the promise of the 2000s but that this is most likely due to experimental constraints on datasets, and the success of BNs at inferring networks in a variety of biological contexts suggests they are a powerful tool for biologists.

## 1. Introduction

Bayesian networks (BNs) are a method of network inference that saw great interest in biological applications around the turn of the millennium [1–3]. Since then, BNs have been applied to many biological disciplines to infer inter-variable dependencies, such as gene regulation, neuronal circuits and interspecific interactions [4–6]. However, despite this interest, there has yet to be a review of BNs’ efficacy at solving biological problems: how well have BNs performed over the past two decades, and what questions are they suited for? Here, we address these questions with a review of the use of BNs in biological applications, and we highlight open questions regarding their efficacy for biological problems.

Mathematically, a BN is a factorization of a joint probability distribution into an acyclic set of conditional dependencies, graphically represented as a directed graph (see Box 1) [7]. Each such factorization can be evaluated through an estimate of how well the structure represents dependencies within the data, typically termed a network ‘score’. Such a score can be used to find an optimal network structure or the network that best fits the data (see Box 2) [9].

Box 1: **Bayesian network basics**

**Figure 1.**
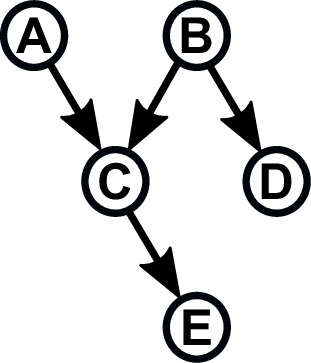
A BN. The circles are the nodes or variables, and the arrows represent direct statistical dependence between nodes. In this network, nodes A and B are parents of C; node D is a second child of node B and node E is a child of node C.

A Bayesian network (BN) is a graphical representation of statistical relationships among a group of variables. BNs are presented in the form of **nodes**, representing variables and shown as circles or other shapes and **links** (sometimes called arcs or edges), representing direct statistical dependence between variables and shown as arrows. Such a representation is called a **graph**. Relationships among variables in the network are described by a family analogy. The node to which an arrow points is called a **child**; the node at the base of the arrow is called its **parent** (figure 1). Parent–child relationships are those of **conditional probability**: the value of a child node depends on the values of its parents.

This graphical representation showing connections between variables relates directly to the mathematical equation for their **joint probability distribution**. If we consider our variables to be $X_{1},...,X_{n}$, the joint probability distribution $P(X_{1},...,X_{n})$ represents the probability of all the variables being in any of their possible states. It is equal to the product of the conditional probability of each individual node given its parents:

$$P(X_{1},...,X_{n})=\prod_{i=1}^{n}P(X_{i}|Pa(X_{i})),$$

where $Pa(X_{i})$ represents the collection of parents of $X_{i}$ and $P(X_{i}|Pa(X_{i}))$ represents the conditional probability of node $X_{i}$ given its parents.

Because BNs are related to the joint probability distribution in this way, they are restricted in that the network cannot have any loops. This is due to mathematical rules about how the joint probability can be constructed from constituent conditional probabilities: it must always narrow down to the probability of a single variable at some point, and cannot loop back on itself. For this reason, BNs are known as **Directed Acyclic Graphs** or **DAGs**.

Box 2: **Learning the structure of Bayesian networks**

BN structure learning is the process of learning the structure—the collection of links between nodes—of the graph representing a BN, using data observed from a system. There are two main approaches to structure learning, constraint-based and score-based. Constraint-based algorithms use conditional independence tests to identify which variables are statistically independent and link those that are not.

Score-based algorithms are more common [8], and thus will be covered here in more detail. Score-based algorithms are based on Bayes’ rule, which relates conditional probabilities to each other, looking at the probability of a graph, G, given the observed data, D:

$$\begin{array}{c} P(G|D)=\frac{P(D|G)P(G)}{P(D)}. \end{array}$$

This probability is typically handled in logarithmic form, due to the very small nature of numbers involved when calculating probabilities across a large number of possible graphs and datasets:

$$\begin{array}{c} \log(P(G|D))=\log(P(D|G))+\log(P(G))-\log(P(D)). \end{array}$$

In practice, the log probability of the data is not calculated, as when searching for a network structure to describe a dataset, this value is constant across all structures; thus, it does not impact their comparison. The remaining values, log⁡(P(D|G)) and log⁡(P(G)), are known as the log **marginal likelihood** of the data given the graph and the log **prior probability** of the graph, respectively. In many cases, the log prior probability of the graph is not calculated, either being considered equivalent across all structures and thus similarly unnecessary to calculate as the probability of the data, or alternatively what is known as a ‘hard prior’ is used. In a hard prior, some links are considered either required or disallowed. All graphs violating these strictures are assigned a probability of zero and thus not considered, and all the remaining are considered equally probable and again the value is not calculated for comparison. Alternatively, log⁡(P(G)) can be used to incorporate prior information about network structure, such as known transcription binding sites.

This leaves calculation of the log marginal likelihood, log⁡(P(D|G)). A variety of different scores exist to calculate this value, either exactly or through an approximation, and finding the structure of a BN reduces to optimizing the value of this score.

Because there are too many possible networks to look at all of them in turn to find the highest score, this optimization takes the form of a heuristic search. A heuristic search is a method of exploring what is known as the **search space** of network structures, using a set of guidelines or heuristics, to guide how the space is explored and how to choose a highest, or at the least a very high, scoring network.

**Figure 2.**
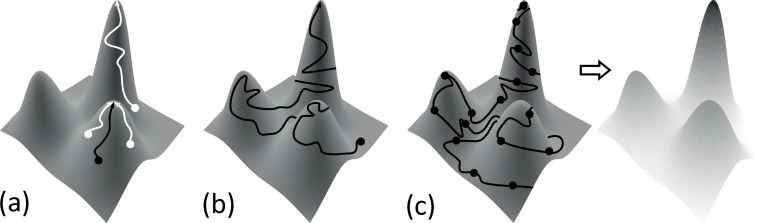
Heuristic searches. A conceptual ‘search space’ is represented by a surface where height represents network score and where adjacent points on the surface correspond to networks separated by a single change (note this is conceptual only, an actual network search space has too many dimensions to plot in this way). (a) Greedy search. The search begins at a randomly chosen network (black/white dot). Only changes which increase the score are allowed (following the lines), and the search completes when no change further increases the score (location pointed to by arrowhead). Greedy searches can get stuck in lower optima, for example, the black arrow terminates on a low peak; thus, multiple searches (e.g. white arrows) are often carried out to explore more of the search space. (b) Simulated annealing. The search begins at randomly chosen network (black dot), and changes that increase and decrease the score are allowed (following the line). Changes that decrease the score become less probable as the search continues, until the end stage replicates a greedy search and a single network at the top of a peak is identified (pointed to by arrowhead). (c) MCMC. A search wanders through the search space (left: following the line) sampling networks at regular intervals (black dots). Locations of the search space are visited proportionally to their probability, such that the collection of samples contains more high-scoring networks (right: darker shading indicates more samples). A process of model averaging from these samples provides probabilities for any particular link being in the solution.

The simplest heuristic search is known as a **greedy search** or **hill-climbing** (figure 2a). This search proceeds in steps, with each step being a different network structure. The search starts at a random location, i.e. a randomly selected BN, where each link is present or not based on some pre-defined probability. The score of this starting point is noted. Then, a single change is made: the addition, deletion or sometimes reversal of a link (e.g. the parent becomes the child). If the score of this changed network is higher than the starting point, the search takes its first step on to this new network. This process is repeated, with potential changes evaluated and steps on to new networks taken each time the score of the new network is higher. The search ends when every possible change is evaluated and none produce a better score. This network is known as the **top network** or a **peak** in the search space.

Greedy searches have the potential to get stuck in what are known as **local optima**, since they can only go up—it may be a higher score could be reached if only a different area of the search space was explored. This can be combated by doing many greedy searches with many random starting points, in an attempt to cover more of the search space.

A more dramatic method of avoiding local optima is a different but related kind of search, known as **simulated annealing** (figure 2b), where steps *down* in probability are allowed, to enable exploration of more of the search space. The steps down are taken with a probability based on a combination of the drop in score and a ‘temperature’ parameter (higher temperature leads to higher probability of stepping down); steps up are always allowed. The temperature slowly reduces over the course of the search, such that by the end only steps up are taken and a final peak is reached. Tuning of this temperature parameter is required and can be a complex process.

A last type of search avoids the problem of local optima all together, by providing a probability distribution of links being in a network solution rather than a single network: this is known as **Markov chain Monte Carlo** or **MCMC** (figure 2c). In MCMC, the search space is explored one step at a time as in the other searches, but the goal is to gain a picture of the probability space rather than find a top network. Steps are taken probabilistically based on the change in network score, such that networks are visited in proportion to their score (e.g. higher scoring networks visited more). A collection of sample networks, and their score, is taken during this process. After a period known as ‘burn-in’, where the distribution stabilizes (‘convergence’), then the probability of each link being in the network can be calculated from a process of ‘model averaging’ applied to the sample of collected networks and their scores. Again, tuning of parameters in MCMC is required to achieve convergence within a reasonable period of time.

However, there are several caveats to this method. The first is that the problem of finding the optimal network structure is computationally intractable [10], and so heuristic search methods are needed in practice to find locally optimal networks that may or may not be equivalent to the globally optimal network structure. Additionally, to ensure performant heuristics, the data must either be discretized into a small number of states, or if one is willing to assume interactions are additive, data can be continuous and normally distributed [8]. The second is that as the factorization of conditional probabilities is minimal, BN structures are restricted to being acyclic [7]. This means that a single BN cannot represent a network containing feedback, which is a problem when hoping to infer feedback-rich biological networks [11]. The third and final caveat is that BN scores cannot distinguish between Markov-equivalent structures, i.e. scores cannot distinguish between networks that share the same topology but differ in the direction of their interactions. This makes inferring the direction of an interaction (and thus causality) difficult. While one can unambiguously infer the direction of interactions by making assumptions about the distribution of the data [12,13], these latter two issues (acyclicity and causality) can be overcome using dynamic Bayesian networks (DBNs), which preserve the acyclic structure of a BN but unfolded through time, allowing inference of a cyclic structure (see Box 3). Here, one can overcome the issues posed by Markov equivalence by only considering dependencies that move forward in time.

Box 3: **Dynamic Bayesian networks**

**Figure 3.**
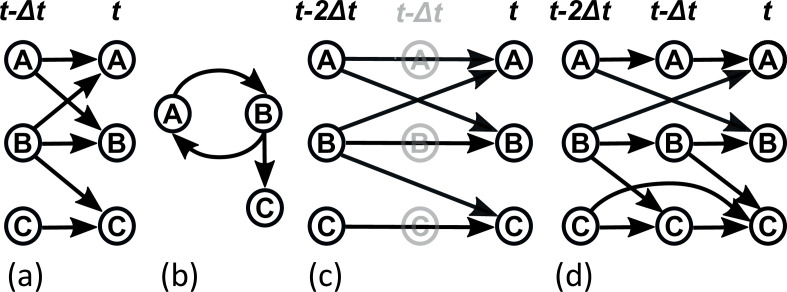
DBNs and Markov lags. (a) A simple DBN with three nodes, A, B and C, and a single Markov lag. In this network, all variables are predicted by themselves in the previous time step. A and B predict each other with one Markov lag (one time step), and B predicts C with one Markov lag. (b) The DBN in (a) shown collapsed over time: A and B form a loop and B is connected to C. (c) A DBN with Markov lag of 2. This network has a similar structure to (a), except that the influences are over a longer period of time. All variables are predicted by themselves two time steps in the past; A and B predict each other with a Markov lag of 2, and B predicts C with a Markov lag of 2. (d) Multiple Markov lags in one network. Again, the structure is similar to (a), differing only in time dependence. All variables are predicted by themselves one time step in the past; C is also predicted by its own value two time steps in the past. A and B predict each other with a Markov lag of 2 (over two timesteps); B predicts C with a Markov lag of 1 (one time step).

The fact that Bayesian networks are DAGs means that it is difficult for them to model systems with feedback loops. However, when data samples are taken as a time series, feedback can be modelled by incorporating time into the network through a **dynamic Bayesian network** (**DBN**). A DBN is structured in time slices, which includes every variable; the simplest DBN including only two time slices, representing two points in time: time t−Δt and t, where Δt represents the interval between samples in the time series (figure 3a). Links are restricted to go only forward in time, from variables in slice t−Δt to those in slice t. In this way, loops can be formed over time (see figure 3b).

DBNs are characterized by how many time slices they have and how these slices relate to the interval between samples in the time series. In the simplest DBN, there are two time slices and they are separated by Δt, equivalent to the sampling interval in the data. Such a DBN has a **Markov lag** of 1: only one point in time, one sampling interval in the past, is required to predict the values of variables in time t (figure 3a). More complex DBNs can have longer Markov lags, e.g. a Markov lag of 2 would mean that variables in time t−2Δt, or two sampling intervals in the past, are used to predict the value of variables in time t (figure 3c). A DBN can include multiple Markov lags, for example, figure 3d shows a network with Markov lags 1 and 2.

There is currently little consensus within the field as to how best to handle these methodological constraints when inferring network structures from biological data. Indeed, the best approach is often highly circumstantial and dependent on the dataset, in part due to the capability of BN structural learning to incorporate expert knowledge. In the following three sections, we review the use of BNs across molecular biology, neuroscience and ecology, three fields in which BNs have found most use, concentrating on those examples where it is possible to evaluate the accuracy of network recovery. We attempt to evaluate the efficacy of BNs in these fields, particularly with respect to these constraints, as a means to suggest the best approaches in applying BNs to learning network structures from biological data. We finish by summarizing the progress of BNs in biology over the past two decades.

## 2. Molecular biology

Molecular and cell biology is the most prolific field for the application of BNs within biology. Here, authors most commonly use large gene expression datasets, such as microarray or RNA-sequence (RNA-seq) data, to infer large networks of gene regulation [4,14–25]; however, other datasets are also studied, such as flow cytometry and western blot data to infer protein–protein interaction networks [26–29], or chromatin-binding profiles to infer chromatin-targeting interactions between binding proteins [30]. BNs have also been used in large multi-omics studies to reveal dependencies between variables [31,32], rather than to reveal the structure of biological processes. While this is an important area for the application of BNs in biology, such networks are difficult to falsify experimentally and as such we have focused the text here on networks of biological processes, which are amenable to perturbation, and have a large body of literature assessing the efficacy of BNs on experimental data.

BNs were first applied to biological data by Friedman *et al*. [4], who used them to reconstruct gene regulatory networks from microarray gene expression data in yeast [4]. While the inferred network contained many more edges than is feasible to experimentally characterize, the network agreed with the expected hierarchy of regulation in the system, and recovered experimentally characterized dependencies between genes [4]. This, combined with the fact that the authors did not specify any prior knowledge in their learning of the BN, suggested that BNs were an effective tool at reconstructing biological networks from data.

This efficacy was later explicitly tested by Sachs *et al*. [27], who found that BNs correctly recovered an experimentally characterized protein–protein interaction network from flow cytometry data [27]. The success of network recovery in this study was in large part attributed to the high quality of dataset which contained thousands of data points, measured the state of the network in single cells and included perturbational data [27]. The authors illustrated the importance of these factors by showing that removing perturbational data from the analysis significantly impacted inference accuracy, and averaging data from multiple cells, as is done in experimental methods like microarray analysis or bulk RNA-seq, was found to also negatively impact BN accuracy, even when thousands of averaged datapoints were used for BN learning [27].

These conditions are rarely met in biological datasets, particularly in microarrays, where the number of analysed genes can far exceed the number of observations [33,34]. As the predominant interest of BNs in molecular biology is gene regulatory network reconstruction, microarrays have been a favoured dataset for BNs over the past two decades, with numerous examples [16–19,23,24,29,35–40], including some which have revealed novel gene regulatory interactions [19,36,37]. This is surprising, as BNs typically employ heuristic searching to find optimal structures and would be expected to under-perform relative to other methods on large systems with many variables and many possible interactions [41]. Similarly, novel regulatory interactions have been uncovered in ‘gene-rich’, ‘observation-poor’ datasets such as chromatin-binding profiles [30,42], or bulk RNA-seq [14,22,25], which suggests that BNs are a robust method for discovering new regulatory logic in such large biological datasets.

Indeed, some authors have used BN structural learning to discover new components of a network rather than the overall network structure, utilizing the ability of a Bayesian scoring metric to incorporate prior information on network structure. Here, a known, incomplete, network architecture is used as a prior and, using data, new nodes are iteratively added to the network depending on how much they improve the network score. Typically, the node that most improves the network score is chosen at each iteration. Carrying on in this manner, this ‘BN + 1’ algorithm constructs a new structure around the core initial network. This method has been used successfully to identify components of gene regulatory networks in *Arabidopsis* [38], new components of the *Escherichia coli* ROS pathway [37], and new components of the PKA pathway in *Dictyostelium* [36].

In general, the complexity of fitting a BN structure to data is determined by the number of variables and also (specifically in the case of discrete BNs) by the number of discretization levels used, as more levels lead to more possible networks and a larger search space. As anecdotal evidence of how the latter may influence success in learning BN structures, we note the success of van Steensel *et al*. [30] in learning BN structures from chromatin-binding profiles [30]. Here, the authors sought to infer a network describing cross-regulation of chromatin binding by chromatin-binding proteins. Comparison of the inferred network with databases and literature found agreement with many of their inferred edges and negatives, as well as a novel chromatin–chromatin targeting interaction, which they then confirmed experimentally [30]. As the number of proteins considered in the network was relatively small (43), the authors suggest this may have improved the success rate of their BN learning, by decreasing the size of the space of possible networks [30]. However, we also note that they used a two-state data discretization (proteins are either bound, or not), which may have contributed to the authors’ success, by further decreasing the size of the search space. Consideration must also be given to the algorithm used in discretization of the data, so as not to destroy statistical signals present within the data [43].

Another factor that must be addressed when handling molecular data is the acyclic nature of BNs, which has been implicated in increasing error rate in feedback-rich biological systems [27]. Using DBNs to overcome this limitation is tricky however as high-dimensional time series of gene expression are technically challenging to collect. Studies can use pseudo-time series of gene expression for learning DBN structures [17,18,21,26,29,35,39,44]; however, this could in principle introduce noise from treating multiple independent measurements as a dependent time series of measurements. Cantone *et al*. and Hill *et al*. studied DBN efficacy in molecular studies of pseudo-time series measurements [26,44]. In both studies, the ground truth of the network structure was known, allowing network recovery to be assessed empirically [26,44]. Neither study suggested that DBNs were effective at inferring the global network structure, but they were successful in revealing a previously uncharacterized interaction between the MAPK and STAT pathways in the Hill *et al*. dataset [26,44]. Additionally, for one of the two datasets studied by Cantone *et al*., the inferred DBN structure was more related to the true structure than would be expected by a null model assigning random edges between genes, suggesting a degree of success at network reconstruction [44]. Interestingly, this dataset had a smaller time interval between samples than the second, worse-performing, dataset analysed by Cantone *et al*., with samples being taken every 10 min rather than every 20 min [44]. While the authors suggest the difference in performance between the two datasets could be due to fewer time points being taken in the 20 min dataset [44], it may also be explained by regulatory interactions taking place at sub-20 minute timescales. It is well known from simulation studies that DBN learning performs poorly on datasets where the timescale of interactions is smaller than the time interval between samples [45,46]. Thus, by sampling more frequently the quality of network recovery may be improved, although the data are sampled at intervals much less than the time scales of the interaction dynamics a variable Markov lag may have to be introduced (see Box 3) [45,46].

Although BNs are clearly an effective tool for the discovery of regulatory interactions, we still lack a clear picture of their efficacy in the global network reconstruction, when applied to non-idealized biological datasets, such as microarray data. A contributing factor to this is that despite semi-frequent experimental testing of edges in learnt BN structures, false positives are rarely reported, and therefore, we lack a comprehensive view of BN error rate outside of a few select examples [27,47]. While it is possible that this may reflect high fidelity of BNs in network reconstruction, we cannot rule out that negative results are going unpublished, and therefore suggest that authors make an effort to communicate these results if they occur. Conversely, the absence of false positives in the literature may reflect the choice of interactions tested, as some authors test only the most connected genes in a network as putative ‘master regulators’ [14,19,20,22], and therefore the statistical signal of these highly connected nodes may be higher within the dataset and less prone to error. Whatever the reason, the lack of a clear error rate across the literature stands as a major obstacle in our understanding of BN efficacy and further work is needed in this area.

## 3. Neuroscience

Following their success in molecular biology [1], BNs were applied to neuroscience, with the first applications being a series of independent publications in 2005 and 2006 [5,48,49]. Here, BNs are typically used to infer the information flow between neurons or regions of the brain, by learning a BN structure from neural activity data, such as electrode measurements or fMRI [5,50–52].

BNs have proven to be a successful method in neuroscience, with many applications [53]. Some studies have focused on using BNs to reconstruct networks with biological relevance, for instance revealing the flow of auditory information in songbird brains, or how neural interactions change depending on whisker stimulus and sensory deprivation in rats [5,50,54]. Many others have focused on reconstructing networks with a clinical application, for instance examining differences in effective connectivity in schizophrenia, or understanding changes in effective connectivity during visual recognition [55–57]. One study focused on using learned DBN structures to recognize left and right movement patterns in EEG data from patients with motor disabilities [58].

For electrophysiological data, DBN structural learning is known to perform very well, successfully recovering a network of information flow for regions of songbird brain in response to auditory stimuli and suggesting novel routes of neural information flow [5]. The performance was evaluated by comparison with known neuroanatomy, taking into account positioning of the electrodes, and by the fact that edges were consistently recovered across multiple datasets for each bird in the study, suggesting the algorithm recovered biologically meaningful interactions [5]. As the authors were able to dissect their specimens and assess the network structure based on electrode positioning, this study served as the first demonstration of BN efficacy in learning neural circuits [5]. Similar results were reported for rat whiskers [54]. The success of DBN inference in such datasets agrees with the intuition outlined above as the number of data points often far exceeds the number of variables, the data is a true time series, and measurements are made at a very fine temporal resolution.

The efficacy of BNs at learning structures from other data types such as fMRI or EEG is less clear, as a large body of this literature focuses on human subjects, where the ground truth of the network is more difficult to establish. However, there is a plethora of evidence to suggest that BNs are successful, for instance, DBN structures learnt from fMRI data possess many edges that would be expected from prior literature [51], and BNs have been shown to be highly successful at inferring structures from synthetic fMRI data [59]. The efficacy of BNs in learning structures from EEG data is less clear and further work may need to be performed here, but the fact that DBN studies can recover biologically meaningful differences in network structure suggests that the method may be at least partially successful in learning the true structure [55,58].

## 4. Ecology

Ecology is the third major area of biology in which BNs are used. Here, the aim is almost unanimously to learn the biotic interactions between taxa from nothing more than count data or relative quantities of taxa [6,60–70]. Compared with the other fields discussed, ecology is the newest field for BNs, with the first application in 2010 studying upland bird communities [6]. BNs have proven to be a useful tool for inferring ecological networks, and identifying species of conservation interest. For instance, Pozsgai *et al*. identified highly connected species within a BN structure as candidate indicator species for streamlined ecological monitoring [60]. Similarly, BN structures of deep-sea sessile fauna revealed Porifera as a keystone group with the community [64].

Unlike molecular biology or neuroscience, however, there has yet to be an explicit demonstration of BNs’ efficacy in global reconstruction of ecological networks. Sander *et al*. [62] attempted to evaluate the performance of DBNs on species presence/absence data based on two datasets with known underlying biotic interaction networks; however, they did not find DBNs to be effective at recovering these structures [62]. Rather, they found that DBN structures were only non-randomly related to the true structure when studying the non-trophic interaction network of an intertidal community, and marginally significant when studying the trophic network of a freshwater fish community [62].

However, there are examples of BNs successfully inferring ecological interactions. For instance, BN structures have been shown to recover interactions between upland bird species in agreement with field observations, recovered expected interactions in a simple rocky shore ecosystem, and DBN structures can be recovered that are non-randomly related to an underlying species–species interaction network [6,62,67]. Additionally, incorporating learnt BN structures representing species–species interactions into species distribution models can greatly improve the model’s predictive performance compared with when only environmental variables are considered [71,72]. This is consistent with BNs recovering true species–species interactions, but we cannot rule out the possibility that they are detecting correlations in species abundance reflective of variations in abiotic factors below the resolution measured in the data [71]. It is, therefore, likely that BNs are at least marginally successful in recovering ecological networks, much like their performance in microarrays or bulk RNA-seq.

Additionally, there may be factors at play that contributed to poor network structure recovery in the work of Sander *et al*. DBNs performed successfully on a non-trophic network structure for the intertidal community but failed on the trophic network structure for the same dataset, and only recovered a marginally significant structure for a trophic network of freshwater fish [62]. While the authors did not have another non-trophic network for comparison, they proposed that the two-state discretization method used was too coarse to reflect the dynamics of trophic interactions, and thus partly to blame for poor inference [62]. While this is likely true, it is also possible that the 1 or 2 year interval between samples was much greater than the true timescale of the dynamics and therefore decreased the performance of DBNs [45,46]. An interesting comparison would be inferring BN structures on the datasets used by Sander *et al*., and seeing if BNs are more effective than DBNs at recovering the underlying network. Additionally, these results may reflect weaknesses in the search technique. For example, ecological datasets have been shown to have multiple local optima and minima under the Bayesian Dirichlet equivalent (BDe) score, as used by Sander *et al*. [6,62,66]. Such a shape to the search space will significantly impact the performance of score-based heuristics. The fact that only DBN structures with the parent count restricted to 2, rather than 3, 4 or 5, were successful in Sander *et al*.’s study may also hint at search space determining method performance. Specifically, by reducing the number of parents permissible per node the dimensionality of the search space is significantly reduced, and so by restricting this, optimal peaks are more discoverable by the searcher. Further evidence for this being the case comes from correlation-based methods performing successfully on all datasets, as correlation methods have been shown to out-perform BNs for large search spaces, due to BNs’ dependence on heuristics [41].

The use of BNs is not restricted to macroscopic ecology, and BNs have proven useful in a variety of applications within microbial ecology. For instance, BNs have been used to construct community networks to explain the pathobiology of oak mildew, or dynamic changes in the microbial composition of gravesoil, using abundances of operational taxonomic units (OTUs) in metagenomic data [61,70,73]. Additionally, BN structures may be used to study medically relevant microbial communities, such as the oral and gut microbiome, and their response to infection [74–77]. While the accuracy of these networks is unclear due to the difficult nature of verifying such interactions, again anecdotal evidence points to some degree of network recovery. For instance, a DBN structure was able to correctly predict dynamic changes in the gut microbiome of infants, suggesting the inferred structure is at least capable of predicting the true dynamics [75]. As this hints, BN structures are also useful in predictive models of microbial ecology, specifically predicting the dynamical community composition over time. Larsen *et al*. pioneered this by integrating a learned BN community structure with an artificial neural network (ANN) to predict marine microbial community make-up based on physical factors in the English Channel [78]. Their approach was highly successful, and has inspired a wide variety of similar studies on predicting community composition [79–81], and even changes in gene expression in Aspen trees in response to pollution [82]. As similar approaches have been shown to be successful in macroscopic ecology [71,72], it seems likely that over the coming years we will see BNs increasingly used as a means to integrate community interactions into predictive models.

## 5. Conclusions and outlook

Overall, BNs are successful in learning biological interdependencies from data, correctly inferring relationships between variables from data in all fields examined. However, we still lack a clear understanding of how effective they are in global network recovery. Thus far, the examples of successful network recovery by BNs are on systems involving few variables and with few (2–3) discretization states, agreeing with theoretical intuition (Box 2) and simulation studies [45,83]. However, as to assess network recovery one must in general (unless one is willing to aggregate the results of multiple learning methods [84]) know the true structure *a priori*, this analysis is for now limited to small, experimentally tractable networks and BNs may be effective on a broader range of systems than suggested here. This is something that will only become clear as our experimental understanding of biological systems improves.

We note however that even when the globally recovered network is incomplete, the ability of BNs to discover highly nonlinear and combinatorial relationships from data [8] has been highly effective at discovering dependencies previously undetected by other statistical methods. Additionally, the ability to integrate prior knowledge about a network structure has been extremely useful when trying to discover new components of a network, as in the case of the ‘BN + 1’ studies discussed above [36–38]. Therefore, despite present uncertainty about the fidelity of BNs in global network recovery, there is ample evidence that BNs are a powerful and appropriate tool for biological network inference, at least at the scale of local interactions.

DBNs appear to be very effective for inferring structures in neuroscience, and electrophysiological data appears suited to DBN analysis with mixed Markov lags. Where tested, DBNs have performed poorly on molecular and ecological datasets, however, as previously discussed it is unclear whether this reflects a general trend or is merely the limitations of the datasets used. Electrophysiological data are typically characterized by a very high temporal resolution, which may make it more amenable to DBNs. A pragmatic study would be to compare the efficacy of DBNs with conventional BN learning where one is interested in inferring a cyclic network from biological data, as conventional BNs may be more effective for short time series or time series where the time between collecting data points is greater than the true timescale of interactions in the system. We note that there is yet to be an application of DBNs to truly dynamic data in molecular systems, and suggest that the method may be extremely successful in recovering molecular interaction networks from fluorescent reporter datasets. In our view, DBNs continue to hold promise across biology and we encourage their application to novel datasets with high temporal resolution.

It is also worth noting, as discussed above under ‘Molecular Biology’, that false positive links between variables in learnt structures are rarely reported, across all literature on biological applications of BNs. For some fields like ecology or neuroscience this is unsurprising, as perturbational studies verifying or refuting links are often impossible; however, this is often feasible in molecular studies, and indeed authors do use perturbational methods to verify (or refute) links [14,19,27]. As experimentally characterizing ‘gold-standard’ reference biological systems for evaluating performance of network inference is challenging it may be more practical to assess BN efficacy through a community approach, where reports of false positive links give a crude estimate of search accuracy for different systems. We therefore encourage authors to publish false positive results when experimentally testing BN predictions, to aid in a field-level understanding of the performance of the method. However, we note that assessing the rate of false negative or false positive edges in a network may not be the best method of assessing how ‘good’ a BN structure is. The topology of a biological network cannot always predict system dynamics in isolation [85], and in practice different edges of the network may not have equally large contributions to system behaviour. As a result, the definition of the ‘best’ network is likely to be circumstantial. Take, for example, the case of researchers studying a hypothetical gene regulatory network: if these researchers hope to use a BN structure to predict the macroscopic outcome of a perturbation to the network, then the correct inference of weak interactions between genes is unimportant to them. Conversely, if they were interested in the precise gene regulatory architecture of the network, for instance, to predict the evolutionary potential of the network [86], then the fidelity of structural learning is extremely important. Thus, we advocate for further use of BNs in informing experimental studies, to better understand what a ‘successful’ network looks like in different disciplines of biology.

We conclude by comparing the performance of BNs with the promise they showed in the early 2000s as a means to accurately infer complete biological networks from large datasets [1,4]. While BNs and DBNs have been shown to be successful in accurately inferring biological interactions in a wide variety of systems and datasets, they have not risen as a ‘magic bullet’ that can accurately recover large networks from only observational data. However, this shortfall is possibly due to the difficulty of obtaining large many-variable datasets of sufficient quality for BN structure learning, and we also cannot accurately assess their efficacy on large networks as few ‘gold standard’ datasets for such networks exist. Therefore, any failure to live up to this promise may not be a failure of the method *per se*, but rather may reflect the current limitations of experimental techniques or the large sample sizes required by current structural learning techniques. As experimental methods continue to improve and advances are made in structure learning (e.g. such as advances in optimization [87]), these issues may become less limiting. This, plus the evidenced success of BNs in accurately identifying novel interactions, this provides some optimism for the future, and BNs may prove powerful tools for network inference as experimental techniques and structural learning methods improve.

## Data Availability

Supplementary material is available at [88].
